# Non-Contact Translation-Rotation Sensor Using Combined Effects of Magnetostriction and Piezoelectricity

**DOI:** 10.3390/s121013829

**Published:** 2012-10-15

**Authors:** Bintang Yang, Qingwei Liu, Ting Zhang, Yudong Cao, Zhiqiang Feng, Guang Meng

**Affiliations:** 1 State Key Laboratory of Mechanical System and Vibration, Shanghai Jiao Tong University, Shanghai 200240, China; E-Mails: lqwei7@gmail.com (Q.L.); haohaomumu1@163.com (T.Z.); gmeng@sjtu.edu.cn (G.M.); 2 School of Mechanical Engineering, Purdue University, West Lafayette, IN 47906, USA; E-Mail: cao23@purdue.edu; 3 Université d'Evry-Val d'Essonne, LMEE, Evry, 91260 Juvisy-sur-Orge, France; E-Mail: zhi-qiang.feng@ufrst.univ-evry.fr; 4 School of Mechanics and Engineering, Southwest Jiaotong University, Chengdu 610031, China

**Keywords:** displacement sensor, translation-rotation measurement, magnetostrictive sensor, piezoelectric effect

## Abstract

Precise displacement sensors are an important topic in precision engineering. At present, this type of sensors typically have a single feature of either translation or rotation measurement. They are also inconvenient to integrate with the host devices. In this report we propose a new kind of sensor that enables both translation and rotation measurement by using the combined effect of magnetostriction and piezoelectricity. As a proof of concept, we experimentally realized a prototype of non-contact translation-rotation precise sensor. In the current research stage, through both theoretical and experimental study, the non-contact displacement sensor is shown to be feasible for measuring both translation and rotation either in coarse or fine measurement. Moreover, owing to its compact, rigid structure and fewer components, it can be easily embedded in host equipment.

## 1. Introduction

Following the development of the electronic and automation industry, displacement sensors have been widely used and they have become a vital part of measurement technology. Common measurement mechanisms are based on electromagnetism and photoelectricity. They both transform displacement into electric signals. Taking photoelectric angular displacement sensor as an example, the most common methods include optical collimation [1], optical internal-reflection [2], laser interference and circular laser [3]. Each of them can measure a small angle precisely. However, their structures are complex and their sizes are relatively large. In addition, most sensors can only measure linear or angular displacements. Therefore it is necessary to develop a compact, high precision, high resolution, and steady non-contact displacement sensor to meet the demands of real-time measurement. In magnetostrictive displacement sensors [4], the measurement is conventionally realized by monitoring a pulse produced by two intersecting moving magnetic fields. However, on some occasions the pulse may be too small. Also, the induced voltage may have long delay times which causes the short range measurement errors to increase. Another new measurement mechanism that has emerged in recent years is the Magnetoelectric Effect (ME) sensor. The magneto effect is due to the combined property of magnetostrictive and piezoelectric components in the sensor. Because ME enables the high sensitivity transformation from a magnetic signal to an electric signal, researchers have found it an attractive option for developing magnetic field sensors and current sensors [5]. However, to our knowledge there have been no reports on using ME for displacement sensors.

**Figure 1.**
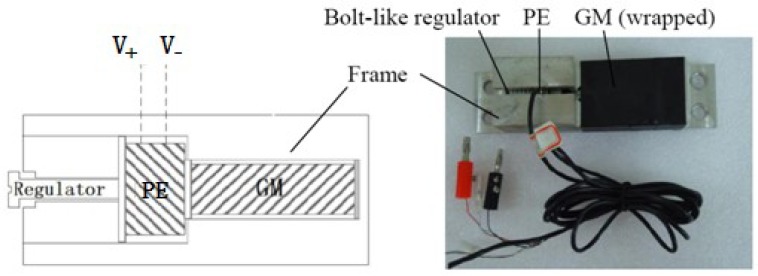
The schematic diagram and the prototype photograph of the non-contact displacement sensor.

**Figure 2.**
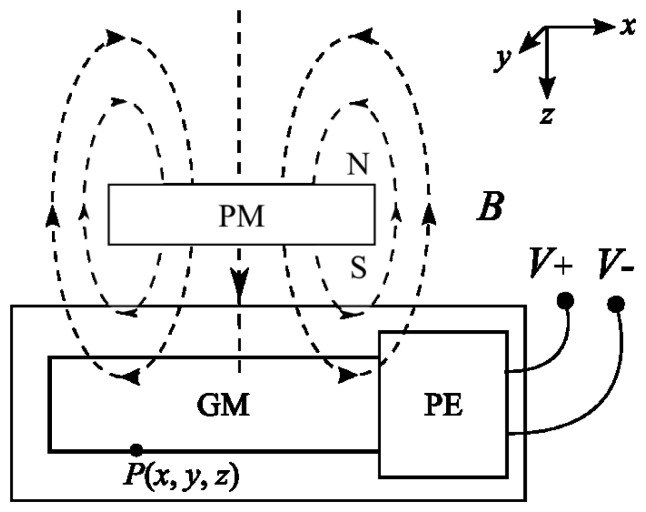
The schematic diagram of the sensing mechanism.

In this work we propose a displacement sensor that takes advantage of both the magnetostrictive effect and the piezoelectric effect [6]. It has a compact size, high sensitivity and a relatively simple structure and assembly. A schematic diagram and a photograph of the prototype are shown in Figure 1, where a block Giant Magnetostrictive (GM) material is joined to a block of Piezoelectric material (PE). They are embedded in a rigid aluminum frame. On the left, next to the PE, is a bolt-like regulator which is used to apply and adjust the prestress values on the PE and the GM, which are optimized based on the bias effect of these two materials [7]. When an external permanent magnet field cuts through the magnetostrictive material and excites the magnetostrictive material to stretch, an induced voltage is produced on both sides of the piezoelectric material component. The principle is illustrated in Figure 2.

The proposed sensor featuring the non-contact and direct measuring principle and simple structure has the unique potential of realizing both the measurement of linear and angular displacement, namely a Non-Contact Translation-Rotation Sensor (Nc-TRS).

The organization of the paper is as follows: Section 1 gives a brief introduction of the non-contact displacement sensor. Section 2 presents a mathematical model for the displacement. Section 3 proposes the experimental verifications, experimental results and some discussions. We conclude the paper in Section 4.

## 2. Mathematical Model

The Nc-TRS's principle used in this study is illustrated in Figure 2. When the permanent magnet (PM) block is moving vertically, the magnetostrictive material cuts through the magnetic field generated by the PM and a horizontal magnetostrictive output force is induced. Supported by the rigid frame, the GM can exert a force on the piezoelectric material (PE). Due to the piezoelectric effect, PE will produce a voltage between its upper and lower surfaces.

Clearly, the Nc-TRS is realized based upon both magnetostrictive and piezoelectric effects [8]. Specifically, in this study the Joule magnetostrictive effect is used. It is a property of ferromagnetic materials which can change in shape and dimensions in the magnetization direction [9,10]. In addition, the electromechanical coupling coefficient (*K*_33_) of common magnetostrictive materials is 0.3∼0.5. For some giant magnetostrictive materials, such as Terfenol-D, *K*_33_ can reach up to 0.75. Owing to their highly efficient responses, magnetostrictive materials have been widely used to measure non-electric quantities since the late 20th century.

Now we will proceed to discuss the mathematical model of the device. In this model we start with the following constitutive equation of magnetostriction:

(1) $$\{\begin{array}{c} ɛ=s\sigma +d_{m}H\\ B=d_{m}^{T}\sigma +\mu H \end{array}$$

where *ε*, *B*, *σ* and *H*, are strain, magnetic flux density, stress and magnetic field respectively, while *s*, $d_{m}^{T}$ and *μ* are mechanical compliance with a constant magnetic field, linear piezomagnetic coefficients and magnetic permeability at a constant stress respectively. $d_{m}^{T}$ is the transposition matrixes of *d_m_*.

Since the GM is constrained by the rigid frame, it is reasonable to assume that the strain *ε* = 0, then:

(2) $$\sigma =-\frac{d_{m}}{s}H$$

Apart from the magnetostrictive effect, we also need to model piezoelectricity. Piezoelectricity is the charge that accumulates in certain solid materials [11,12] in response to applied mechanical stress. For a given external stress σ, the electric displacement of the piezoelectric material is:

(3) $$D_{p}=d_{p}\sigma$$

where *d_p_* and *D_p_* represent piezoelectric stress constant and the contribution of the mechanical stress to the electric displacement, respectively.

Using Equation (3), the gathered charges *Q_p_* in the thickness direction of the piezoelectric material is as given by the following expression:

(4) $$Q_{p}=\iint_{A}D_{p}dA=\iint_{A}d_{p}\sigma dA=\int_{0}^{l}d_{p}\sigma w_{p}dx$$

where *w_p_* and *l* are the width and length of piezoelectric material respectively. The voltage across the thickness of the material is then given by:

(5) $$V=\frac{Q_{p}}{C_{p}}=\frac{-\int_{0}^{l}d_{p}\frac{d_{m}}{s}Hw_{p}dx}{C_{p}}=\frac{-d_{p}d_{m}lH}{sC_{p}}$$

The voltage across the thickness of the material is then given by the *z*-component of the magnetic field strength induced by PM at point P (shown in Figure 2). The magnetic field strength can be written as follows [13]:

(6) $$H=\frac{B_{r}}{\mu \pi }[\arcsin\frac{ab}{\sqrt{\left(a^{2}+d^{2})(b^{2}+d^{2}\right)}}-\arcsin\frac{ab}{\sqrt{\left(a^{2}+{\left(d+L_{m}\right)}^{2})(b^{2}+{\left(d+L_{m}\right)}^{2}\right)}}]$$

where *B_r_* is the magnet remnant flux density, *μ* is the magnet magnetic permeability. 2*a*, 2*b* and *L_m_* are the width, height and length of PM, respectively. The term *d* is the displacement along the *z* direction.

Combining Equations (5) and (6), we obtain the relation between the displacement *d* and the voltage:

(7) $$V=\delta \frac{-d_{p}d_{m}w_{p}l}{sC_{p}}\frac{\gamma B}{\mu \pi }[\arcsin\frac{ab}{\sqrt{\left(a^{2}+d^{2})(b^{2}+d^{2}\right)}}-\arcsin\frac{ab}{\sqrt{\left(a^{2}+{\left(d+L_{m}\right)}^{2})(b^{2}+{\left(d+L_{m}\right)}^{2}\right)}}]$$

where *γ* < l is a constant factor due to demagnetization. δ is a uniformity coefficient for nonlinear properties of GM. And set the uniformity coefficient δ = 1 by the assumption that the field of the permanent magnet is strong enough to thoroughly cover the GM.

For the case where the PM is rotating, Equations (6) and (7) need to be re-written in terms of the measured angle *θ*:

(8) $$H_{\theta }=H\sin\theta$$

(9) $$V=\delta \frac{-d_{p}d_{m}w_{p}l\gamma B_{r}\sin\theta }{sC_{p}\mu \pi }[\arcsin\frac{ab}{\sqrt{\left(a^{2}+{z_{0}}^{2})(b^{2}+{z_{0}}^{2}\right)}}-\arcsin\frac{ab}{\sqrt{\left(a^{2}+{\left(z_{0}+L_{m}\right)}^{2})(b^{2}+{\left(z_{0}+L_{m}\right)}^{2}\right)}}]$$

where *z*_0_ is the distance between PM and GM.

In situations where the sensor is used, a permanent magnet (PM) is attached to the moving body whose motion is being measured and we ensure that the GM is covered by the PM's magnetic field. In this process, when the testing body is moving (either linearly or angularly), the GM will tend to elongate, and if the movement on both ends of GM is restricted, the tension in GM will be transferred to the piezoelectric material to produce an electric charge signal. With proper calibration, we could measure the testing body's displacement by detecting this electric charge signal.

## 3. Experimental Results

### 3.1. Materials and Methods

**Figure 3.**
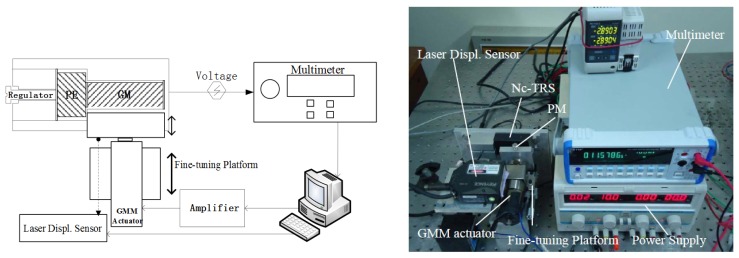
The schematic diagram and the photograph of the experimental setup for displacment measurement.

Based on the above model development, laboratory tests are designed to verify the functionality of the proposed non-contact displacement sensor. With the prototype Nc-TRS, a testing system shown in Figure 3 is constructed. It consists of a GM Terfenol-D rod (*φ*10 × 35 mm), a piezoelectric part (instead of the piezoelectric material, a commercial piezoelectric sensor LC1104 is used for implementing the experiment for convenience at this moment), a giant magnetostrictive material (GM) actuator made in-house, a permanent magnet block (PM, RbFeB-N35, BH_max_/35MGOe, size 10 × 10 × 50 mm), a fine-tuning platform, a laser displacement sensor (Keyence LK-G30), a digital multimeter (NF DM2561, 100 nV resolution) and a power supply. Moreover, the PM is fixed on the output end of the GM actuator and parallel to the GM rod at the same height. They are fixed on the moving table of the fine-tuning platform. The laser displacement sensor points the light on the PM in the movement direction. Using this experimental testing setup, two experiments are specifically designed, which is described in detail below.

In the first experiment, we start by moving the PM with the fine-tuning platform (Figure 3). The displacement of the PM is measured directly by the laser displacement sensor and at the same time measured indirectly through the induced voltage of the Nc-TRS's piezoelectric sensor. The induced sigal is acquired by the digital multimeter. Using the collected data from the both approaches, we can obtain the relationship between the induced voltage and the displacement from 0 mm to 9.7 mm.

Based on this empirical relationship, we can now determine the linear region of the induced voltage as a function of displacement of the PM. Then, we locate the Nc-TRS at the center of the linear region in terms of displacement according to the calibration results. This is equivalent to biasing the displacement. In the meantime, the PM is fixed on the output end of the GM actuator, whose forward and backward movement with respect to the location of Nc-TRS can be precisely controlled by feeding sinusoidal currents into the GM actuator. Consequently, both the coarse movement by the fine-tuning platform and the precise displacement by the GM actuator can be measured by the Nc-TRS and by the laser displacement sensor simultaneously.

**Figure 4.**
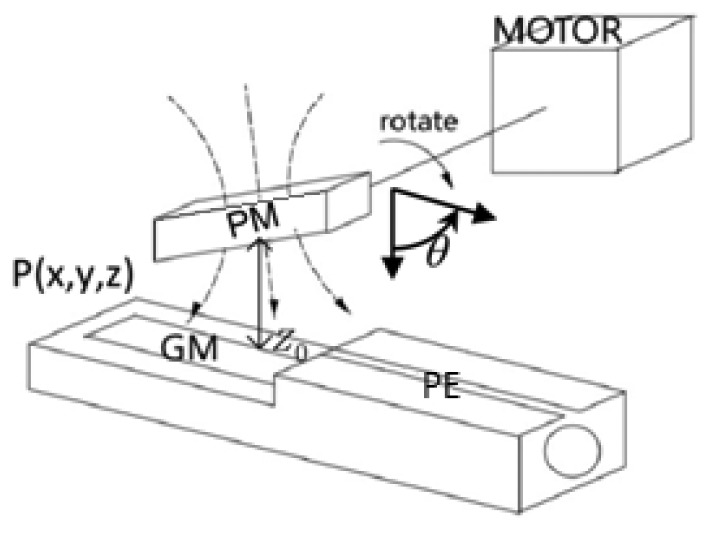
The structure diagram of angle sensor.

**Figure 5.**
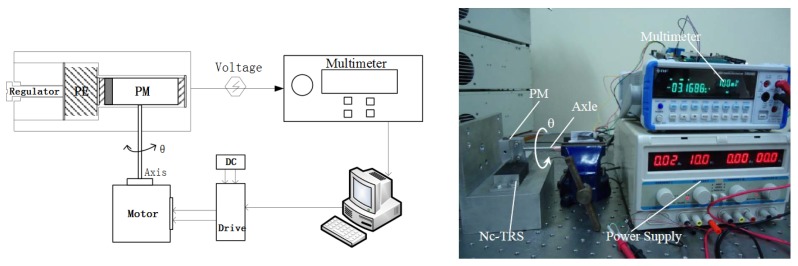
Schematic diagram and photograph of the experimental setup for angle measurement.

The setup for a rotation experimental test is shown in Figure 5. In this experiment, a motor and a motor drive are employed. The PM is fixed on the end of the motor's rotor shaft, and the PM should be located right over the GM rod of the Nc-TRS (see Figures 4 and 5). The PM can be controlled to rotate with the motor shaft. Meanwhile, the GM rod of the Nc-TRS can be stretched alternatively by cutting through the time-dependent magnetic field generated by the rotating permanet magnet. An alternating voltage following the PM rotation is thus induced in the PE. The multimeter then measures the induced voltage as a function of time or a function of rotating angle. In this test, as expected, we have obtained satisfactory results in measuring the angular displacement using the proposed Nc-TRS.

### 3.2. Results and Discussion

**Figure 6.**
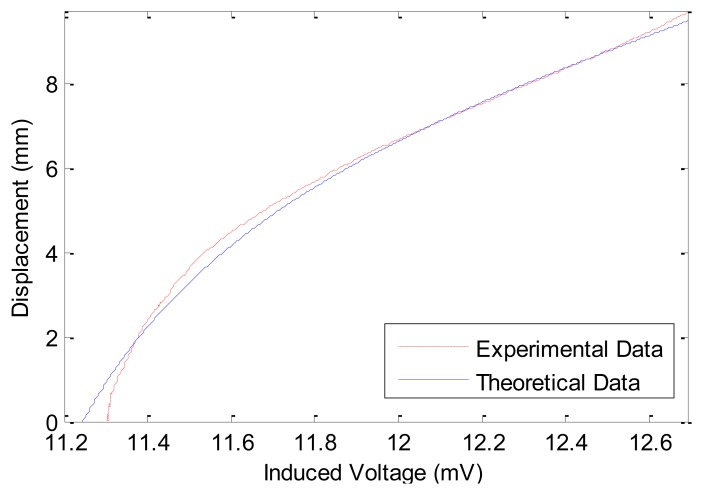
Comparison between the experimental and theoretical results on wide-range measurement.

**Figure 7.**
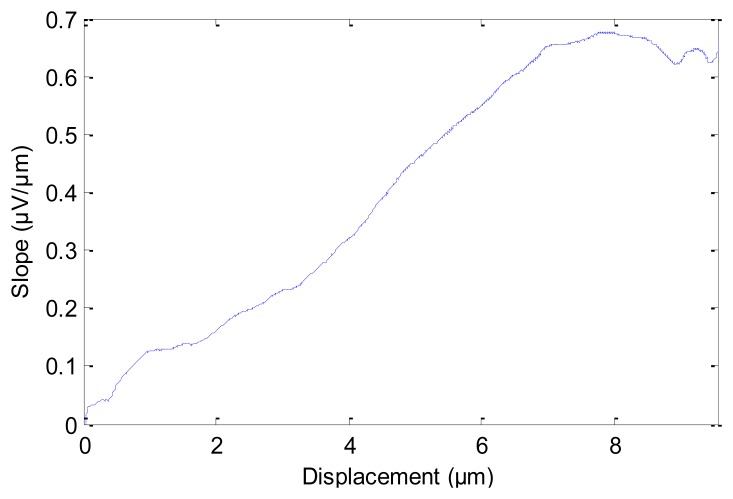
The slope of the curve in Figure 6.

**Figure 8.**
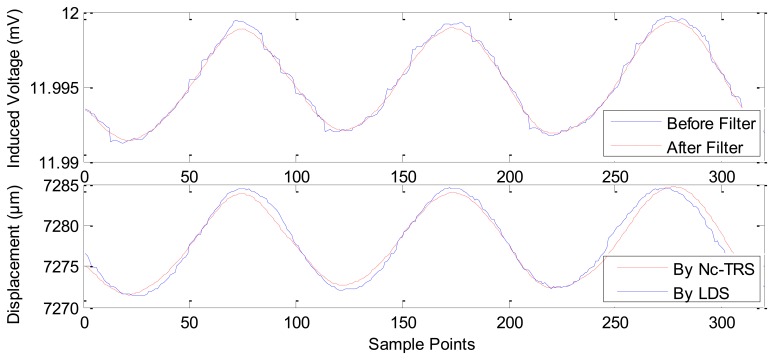
The sensing effect under applied current *I*(*t*) = sin(2π*ft*) A (*f* = 10 Hz).

**Figure 9.**
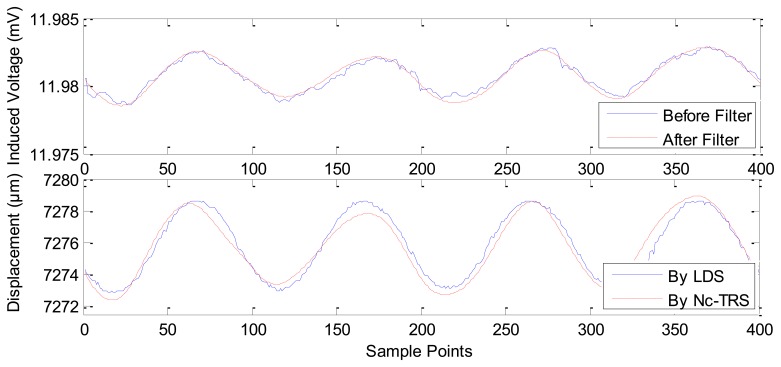
The sensing effect under applied current *I*(*t*) = 0.5sin(2π*ft*) A (*f* = 10 Hz).

**Figure 10.**
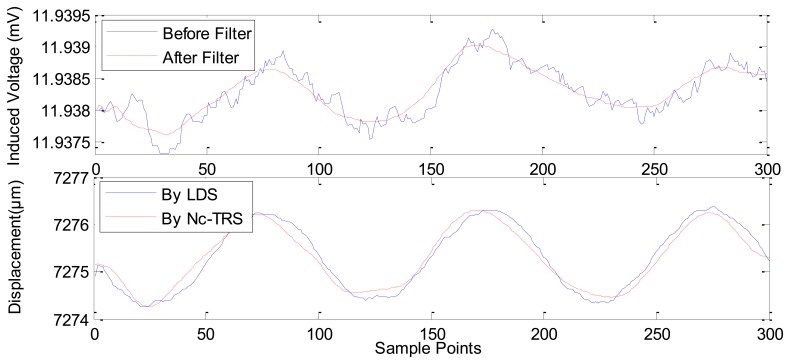
The sensing effect under applied current *I*(*t*) = 0.2sin(2π*ft*) A (*f* = 10 Hz).

**Figure 11.**
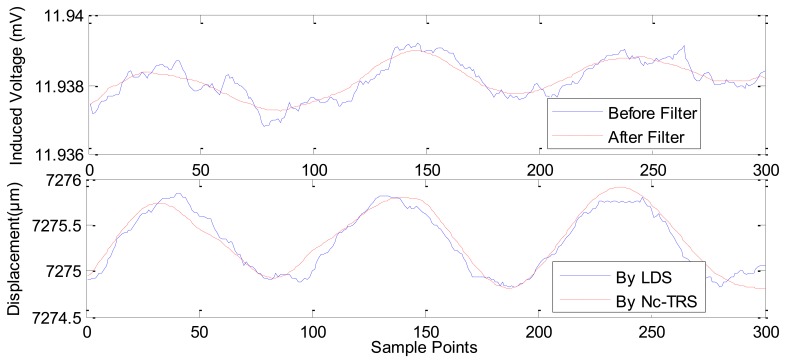
The sensing effect under applied current *I*(*t*) = 0.1sin(2π*ft*) A (*f* = 10 Hz).

**Figure 12.**
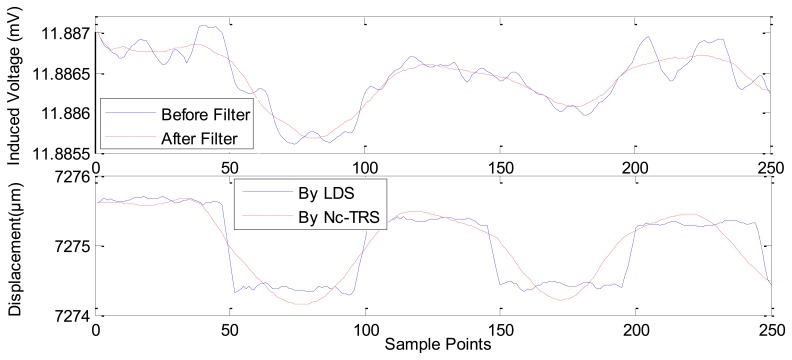
The sensing effect under applied current *I*(*t*) = 0.05square(2π*ft*) A (*f* = 10 Hz).

**Figure 13.**
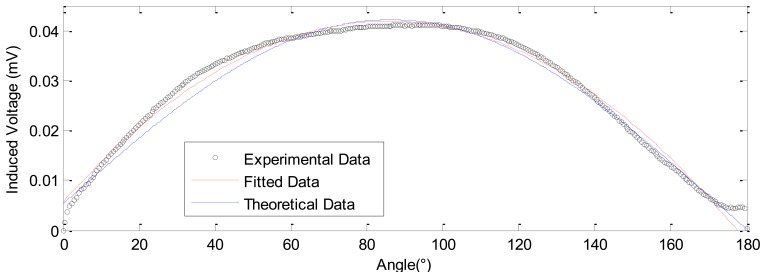
The sensing effect of large angle test.

Using the materials and methods described above, some experimental results were obtained to verify the functionality of the sensor. The experimental tests for measuring large and fine translational and rotational displacement have been performed and the results are the following. Figures 6–13 show the measured curves between the induced electric potential in the piezoelectric sensor and the input moving displacement of PM.

Figure 6 compares the theoretical and experimental results of the calibration process in the first experiment. The theoretical data (solid curve in Figure 6) are calculated using Equation (7). From Figure 6 it is clear that the experimental measurements agree well with the theoretical results. This also ensures that Equation (7) is able to satisfactorily model the relation between induced voltage of piezoelectric sensor and input displacement of PM. In Figure 6, the displacement of PM moves from 0 mm to 9.7 mm, the average slope is roughly 6.7867 mm/mV. From Figure 6 one can observe that as the displacement of PM increases, the induced voltage does not always increase linearly. The linearity property in 0∼2 mm and in 6∼9.7 mm is better than that in the 2∼6 mm range. Correspondingly, Figure 7 shows the slope of the curve in Figure 6 for different values of PM displacements. From Figure 7 it can be observed that the slope from 6 mm to 9.7 mm is approximately constant (about 1/1.578 μV/μm). The slope of the curve in 6∼8.5 mm is steadier than other ranges of PM displacement. Therefore we choose the response curve in 6∼8.5 mm as the optimum range and the point at 7.2745 mm as the central position.

Based on this result, we could locate the point of maximum slope in Figure 7 as the maximum sensitivity point of the Nc-TRS. In this experimental test, the forwards and backwards fine displacement of the PM is provided by the GM actuator under excitation of sinusoidal currents. The experimental results of the fine displacement measurements are plotted in Figures 8–12 and some key parameters are summarized in Table 1.

In Figure 8, the current is applied in GM actuator at frequency of 10 Hz and at amplitude of 1 A. The PM displacement ranges from 7,272.1815 μm to 7,284.2104 μm while the induced voltage piezoelectric sensor ranges 0.0078 mV (from about 11.9917 mV to about 11.9994 mV). Figure 9 shows the range of the PM displacement being 7,272.9158 μm to 7,278.4498 μm when the current applied in the GM actuator is *I*(*t*) = 0.5sin(2π*ft*) where *f* is 10 Hz and *t* is time in seconds. The induced voltage ranges 0.0037 mV (from 11.9789 mV to 11.9826 mV). Similarly, Figure 10 presents the measurement results using applied current *I*(*t*) = 0.2sin(2π*ft*) (*f* = 10 Hz). In addition, Figure 11 gives the experimental results under the applied current *I*(*t*) = 0.1sin(2π*ft*) (*f* =10 Hz). The ranges of displacement in Figures 10 and 11 are 2.1334 μm and 1.2334 μm; the ranges of the induced voltage in Figures 9 and 10 are 0.0014 mV and 0.0008 mV, respectively. In Figure 12, the applied current is square signal with amplitude is 0.05A and frequency of 10 Hz. The moving PM displacement ranges from 7,274.49 μm to 7,275.40 μm, spanning 910 nm. The range of the induced voltage of piezoelectric sensor is 0.0006 mV (from 11.8860 mV to 11.8866 mV).

In Figures 8–12, the Laser Displacement Sensor (Keyence-LG-K30, with 30 nm resolution) and our sensor results are indicated by LDS and Nc-TRS respectively. In addition, the measured displacement by NC-TRS is attained by induced voltage after filtering. Through comparing acquired displacements by LDS and NC-TRS, in Figures 8–12, the average errors of measuring displacement, which are mean values of all points of errors, are 0.3074 μm (2.4%), 0.0757 μm (1.3%), 0. 1334 μm (6.3%), 0.0765 μm (7.7%), 0.0103 μm (1%) respectively. This implies the measurement precision of the NC-TRS is close to the one of the LDS.

**Figure 14.**
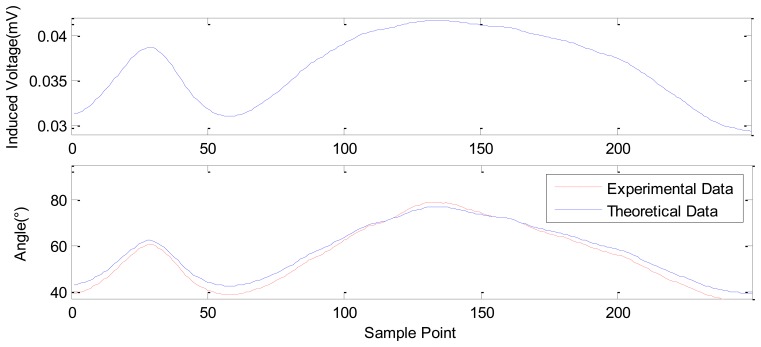
The sensing effect at large angle rotation.

**Figure 15.**
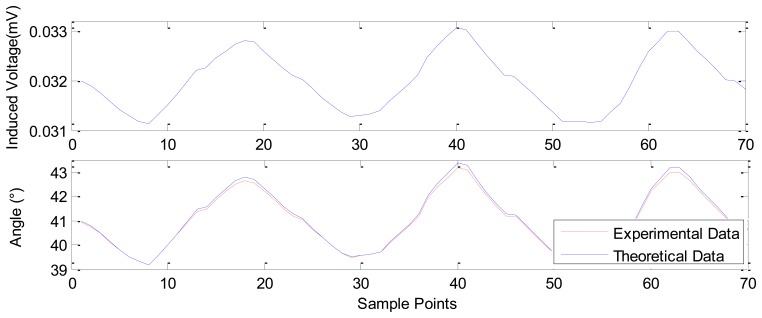
The sensing effect for small angle rotation.

Figures 13–15 show the experimental results of measuring rotation compared with the theoretical calculations based on Equation (9) and the parameters in Table 2. The results prove the validity of the theoretical model in modeling both the large and tiny angular displacement. Moreover, the fitted quadratic polynomial is given, it is as following:

(10) $$IV=(-4.949\times 10^{-6})a^{2}+(8.468\times 10^{-4})a+5.642\times 10^{-3}$$

where *IV* and *a* are induced voltage and rotating angle, respectively.

In Figure 13, the results are obtained for large angle measurement, the range of rotating angle is set as 180°, and the induced corresponding voltage is 0.04113 mV.

Figure 14 shows the results for another large angle measurement. The rotating angle range is set with two different values within 90°. In this figure, the upper subplot describes the induced voltage curve, which is derived from the data from the multimeter. The rotating angle values corresponding to each induced voltage can be solved for using Equation (10). The calculated rotating angles for each data point are plotted as “experimental data” in the lower subplot of Figure 14. The first peak of the curve is at 63.2146°, the 32th sample point and the second peak occurs at 79.7069°, the 143th sample point. The initial angle is 39.2072°. The results shown in Figure 14 also show that the angular measurement is independent of the velocity of angle variation. In addition, the theoretical angle variance based on Equation (9) is plotted in Figure 14 as the “theoretical data”. It also proves the validity of the established model, which can be used to predict the experimental data of angular displacement.

In Figure 15, the results show the sensing property for small angle rotation. The upper curve is the collected induced voltage. In the test, the initial angle of PM is set to be 40°, and the calculated slope at 40° in Figure 13 is 4.8508e−4. Hence, through the slope and the induced voltage in the upper curve, the corresponding angle can be deduced, it is shown in the lower curve indicated with experimental data, where the amplitude is 1.7867° and the set angle is 1.8° (controlled by a step motor in this test).

In summary, by the above theoretical and experimental study, we have realized the large range measurement from 0 mm to 9.7 mm, as shown in Figure 6, and the fine displacement measurements, as shown in Figures 8–12. During the fine measurement process, gradually decreasing displacements are achieved by the GM actuator excited with correspondingly decreasing strength of the current. The Nc-TRS has the potential to improve fine measurement from the micrometric to the nanometric level. Fine displacements of 12.0289 μm, 5.534 μm, 2.1334 μm, 1.2334 μm and the finest of 990 nm has been deduced with respect to 1, 0.5, 0.2, 0.1 and 0.05 A current. The measurement resolution of the designed displacement sensor is close to that of the commercial laser displacement units. In the rotation testing study, referring to Figure 13 to Figure 15, the results show the capability of measuring both large angles from 0° to 180° and small angles around 1∼2° displacement with our sensor, and prove that the sensor may measure angle at the precision of 0.013°.

## 4. Conclusions and Outlook

In the paper, we have proposed a new sensor that is able to measure both translation and rotation displacement by combining magnetostrictive and piezoelectric effects. A non-contact displacement sensor prototype is constructed and tests specifically designed to verify the performance of the sensor are conducted. At the current research stage, through both theoretical and experimental study, the non-contact displacement sensor makes it feasible to measure both translation and rotation either in coarse or fine measurement. The proposed sensor features fewer components, more satisfactory precision and convenience in utilizing, easier assembly into host structures and lower cost compared to other high precision displacement sensors. However, due to the magnetostriction behaviour, the proposed displacement sensor exhibits non-linear properties, such as satuation and hysteresis phenomena. In the future research, we will focus on investigating and resolving these nonlinearities by optimizing the prestress and magnetic field bias. Moreover, by defining the optimal linear-sensening-zone by taking the consideration of both the GM and PE linearity condition, will definitely help to improve the precision and efficiency of the new sensor.

## Figures and Tables

**Table 1.** The analyzed results of the fine displacement measurement.

**Input Current to GM Actuator**		**1 A**	**0.5 A**	**0.2 A**	**0.1 A**	**0.05 A**
Displacement change by LDS (μm)	from	7,271.9333	7,273.05	7,274.4205	7,274.8484	7,274.183
	to	7,284.5333	7,278.6	7,276.247	7,275.8149	7,275.5761
Displacement change by Nc-TRS (μm)	from	7,272.1815	7,272.9158	7,274.2333	7,274.7333	7,274.49
	to	7,284.2104	7,278.4498	7,276.3667	7,275.9667	7,275.40
Induced voltage change (mV)	from	11.9917	11.9789	11.9377	11.9376	11.8860
	To	11.9994	11.9826	11.9391	11.9384	11.8866
Nc-TRS errors by referring LDS (μm)		0.3074	0.0757	0. 1334	0.0765	0.0103

**Table 2.** The related parameters in theoretical simulation.

PE Parameters	
*d_p_* = 2.2 × 10^−11^ *C*/*N*	*C_p_* = 2.8 × 10^−9^ *nF*

GM Parameters	
*d_m_* = 3.92 × 10^−9^ *m/A*	*s_m_* = 3.33 × 10^−11^ *m^2^/N*
*Size: φ*10 × 35 *mm* (Terfenol-D)	

PM Parameters	
*μ* = 0.4	*B_r_* = 1.231*T*
*Size:* 10 × 10 × 50 *mm* (RbFeB-N35)	

Other Parameters	
*z*_0_ = 30 *mm*	
